# Non-verbal nurse-parturient communication in labor in Portuguese-speaking countries*

**DOI:** 10.1590/1518-8345.3032.3193

**Published:** 2019-10-07

**Authors:** Gilmara de Lucena Beserra, Paula Marciana Pinheiro de Oliveira, Lorita Marlena Freitag Pagliuca, Paulo César de Almeida, Saiwori de Jesus Silva Bezerra dos Anjos, Ana Karina Bezerra Pinheiro

**Affiliations:** 1 Universidade Federal do Ceará, Fortaleza, CE, Brazil.; 2 Scholarship holder at the Fundação Cearense de Apoio ao Desenvolvimento Científico e Tecnológico (FUNCAP), Brazil.; 3 Universidade da Integração Internacional da Lusofonia Afro-Brasileira (Unilab), Instituto de Ciências da Saúde, Redenção, CE, Brazil.; 4 Universidade Federal do Ceará (UFC), Departamento de Enfermagem, Fortaleza, CE, Brazil.; 5 Universidade Estadual do Ceará (UECE), Centro de Ciências da Saúde, Fortaleza, CE, Brazil.

**Keywords:** Nonverbal Communication, Obstetric Nursing, Labor, Obstetric, Women’s Health, Natural Childbirth, Nursing Care, Comunicação Não Verbal, Enfermagem Obstétrica, Trabalho de Parto, Saúde da Mulher, Parto Normal, Cuidados de Enfermagem, Comunicación no Verbal, Enfermería Obstétrica, Trabajo de Parto, Salud de la Mujer, Parto Normal, Atención de Enfermería

## Abstract

**Objective:**

to analyze nonverbal communication between nurse and parturient during the active phase of labor in two Portuguese-speaking countries.

**Method:**

a quantitative and analytical study, whose sample consisted of 709 interactions that used the nonverbal communication of nurses and parturients. The analyzed variables were: distance; posture; axis; contact; emblematic gestures; illustrator gestures and regulatory gestures. For the analysis of the data, the Chi-Square and Likelihood Ratio tests were used.

**Results:**

the intimate distance between nurse and parturient in both countries (p = 0.005) prevailed. In both, touch was the most commonly used form of contact (p <0.0001). In both countries, the parturient remained lying down (p <0.0001). In relation to the established contact (p <0.0001), the parturient did not use contact. The face-to-face axis predominated in the interactions in both countries between nurse-parturient (p <0.0001) and parturient-nurse (p <0.0001).

**Conclusion:**

similarities were observed in non-verbal communication between nurses and parturients in both countries. However, there are differences such as the established contact between Brazilian and Cape Verdean nurses to parturients.

## Abstract (pt)

**Objetivo:**

analisar a comunicação não verbal entre enfermeiro e parturiente durante a fase ativa do trabalho de parto em dois países lusófonos.

**Método:**

estudo quantitativo analítico, cuja amostra foi composta por 709 interações que utilizaram a comunicação não verbal dos enfermeiros e parturientes. As variáveis analisadas foram: distância; postura; eixo; contato; gestos emblemáticos; gestos ilustradores e gestos reguladores. Para a análise dos dados, utilizaram-se os testes de Qui-Quadrado e Razão de Verossimilhança.

**Resultados:**

a distância íntima entre enfermeiro e parturiente nos dois países (p=0,005) prevaleceu. Em ambos, o toque foi a forma de contato (p<0,0001) mais usada. Nos dois países, as parturientes permaneceram deitadas (p<0,0001). Em relação ao contato estabelecido (p<0,0001), as parturientes não usaram contato. O eixo face a face predominou nas interações em ambos os países entre enfermeiro-parturiente (p<0,0001) e parturiente-enfermeiro (p<0,0001).

**Conclusão:**

perceberam-se semelhanças nos aspectos de comunicação não verbal entre enfermeiros e parturientes nos dois países. No entanto, observam-se diferenças como o contato estabelecido entre os enfermeiros brasileiros e cabo-verdianos à parturiente.

## Abstract (es)

**Objetivo:**

analizar comunicación no verbal entre enfermero y parturienta durante la fase activa del trabajo de parto en dos países lusohablantes.

**Método:**

estudio analítico cuantitativo, cuya muestra consistió en 709 interacciones que utilizaron la comunicación no verbal entre enfermeros y parturientas. Las variables analizadas fueron: distancia, postura, eje, contacto, gestos emblemáticos, gestos ilustradores y gestos reguladores. Para el análisis de los datos se utilizaron las pruebas de Chi-cuadrado y Razón de Verosimilitud.

**Resultados:**

la distancia íntima entre enfermero y parturienta en los dos países (p=0,005) prevaleció. En ambos, el toque fue la forma de contacto (p<0,0001) más usada. En los dos países las parturientas permanecieron acostadas (p<0,0001). En relación al contacto establecido (p<0,0001) las parturientas no usaron contacto. El eje cara a cara predominó en las interacciones en los dos países, entre enfermero-parturienta (p<0,0001) y parturienta-enfermero (p<0,0001).

**Conclusión:**

se percibió semejanzas en los aspectos de comunicación no verbal entre enfermeros y parturientas en los dos países. Sin embargo, se observan diferencias como es el caso del contacto establecido entre los enfermeros brasileños y los de Cabo Verde con la parturienta.

## Introduction

The process of labor and birth, over the years, has ceased to be an individualized and physiological episode, becoming a process of interventions. In developed countries, childbirth is a process in which interventions are avoided and infrequent. However, there are still health professionals who make the parturition process a traumatic event, failing to establish an approximation with the parturient and ignoring the needs for privacy and comfort in the environment^(^ 1 ^)^.

The birth process in the hospital environment gives new meanings to the birth process. Even with the existence of resources and technologies in these environments, the humanization model in the process of childbirth and birth presupposes that quality care is not synonymous with intervention and technology^(^ 2 ^)^.

In addition to the hospital, there are several environments where parturient care is provided, such as maternities, domicile, birth center and normal delivery center. The performance of deliveries at Normal Delivery Centers (NDC) is associated with lower rates of intervention during labor and delivery, however, gaps in care are still identified, providing women with physical, psychological and socio-cultural changes during labor and delivery that can affect maternal and newborn health^(^ 3 ^)^.

Professionals and women still find barriers to the implementation of more humane and quality obstetric care, such as the structural precariousness of the service, the lack of preparation of women for labor and delivery, and the lack of effective communication among health professionals and the parturient. Through communication based on humanization, information, support, emotional support and affection are made available^(^ 4 ^)^.

In the sphere of nursing, the human being influences the world, is influenced by it and thus interacts with one another. In this sense, it is of fundamental importance, for Nursing care, the communication process, which can be understood as the set of verbal or non-verbal signs or forms transmitted and understood for the purpose of expressing ideas, feelings and thoughts^(^ 5 ^)^.

The human being communicates through verbal communication and non-verbal communication. Not always, non-verbal language is fully understood by professionals working in health services. This type of communication involves the body with its physical, physiological and gestural characteristics^(^ 5 ^)^.

Evidently, the nurse practitioner, who works in the care of labor, must understand non-verbal signals in order to reduce the patient’s anxiety and transmit confidence and security. Non-verbal communication influences both the person receiving the message and the person sending it in different cultural and social settings.

For effective non-verbal communication at the time of labor, the absence of rude treatment, shouting, threats, humiliation and verbal aggression is indispensable^(^ 6 ^)^. However, this may be a reality experienced by many women in Brazil and other Lusophony countries. Thus, it is evident the need to analyze non-verbal communication in this obstetric reality in both lusophone scenarios. At this juncture, the study aimed to analyze the non-verbal communication between nurse and parturient during the active phase of labor in Brazil and in Cape Verde.

## Method

This is a quantitative analytical study, performed in two scenarios (Brazil and Cape Verde): a Normal Birth Center located in a municipality in the metropolitan region of Fortaleza-CE (Brazil) and a maternity hospital located in Praia (Ilha de Santiago / Cape Verde). The data collection period was from March to August 2017 in both countries.

The study population, for both countries, consisted of generalist / obstetrician and parturient nurses. All participants were the senders and recipients observed in non-verbal communication during data collection. It is noteworthy that the analysis of verbal communication has already been described in another study. Parturient included women over 18 years of age, women in the active phase of labor and hemodynamically stable. For the nurses, the professionals who were on duty. Parturients with an indication of possible cesarean section or women at the end of the active phase were excluded. In Cape Verde, parturients who had difficulty understanding and speaking the Portuguese language were also excluded. There was no difference between the inclusion and exclusion criteria of the nurses between the countries, however, the lack of understanding of the Portuguese language was an exclusion criterion present only in Cape Verde for the parturients.

The sample consisted of the number of non-verbal interactions between nurse-parturient and parturient-nurse during the active phase of labor, although the physiological process comprises three phases: Latent Phase, Active Phase and Expulsive Phase^(^ 7 ^)^. The choice of this period was due to the possibility of a long duration, thus enabling the analysis of a larger number of interactions. Altogether, in both countries, 709 interactions were observed.

After explaining the objectives of the study, the Informed Consent Form (FICT) and ethical aspects were met, the data collection instrument was started.

The instrument of data collection to evaluate non-verbal communication was elaborated and validated in a previous study that analyzed the nonverbal communication between the nurse and the person with visual impairment in the Nursing consultation and denominated Non-Verbal Communication Nurse-Blind (CONVENCE). The instrument was validated by three nursing doctoral judges with theses and dissertations oriented on the subject ^(^ 5 ^)^. The instrument was constructed according to Hall’s theoretical framework^(^ 8 ^)^, which is composed of the following variables: nonverbal communication; distance; posture; axis; contact; emblematic gestures; illustrator gestures and regulatory gestures.

The variable non-verbal communication defines the sender, being the nurse, parturient or absent. In cases of lack of interaction, the instrument was marked as “absent”. The distance was classified as intimate (zero to 50cm) and personal (50cm to 1.2m). Regarding body posture, it was analyzed whether the participants, during the interactions, were standing, sitting, lying or kneeling. As for the axis that determines the position of the interlocutors in the communication, they were considered face-to-face, back-facing, socio-fugitive (discouragement in interaction), partner-minded (encouragement in interaction)^(^ 5 ^)^.

The contact variable evaluated the physical contact within a short distance as the touch, caress/touch/grasp, grasp, long hold, located touch, accidentally brush and no contact. The emblematic gestures were characterized as beating the foot and moving the hands, while the illustrated gestures recognize who complements the verbal language or not and the regulatory gestures contemplate the nodding of the head, the movement of the eyes and others^(^ 5 ^)^.

Each instrument was filled in every ten minutes. All moments between the participants in this phase were analyzed. At no time, the researcher influenced the interactions occurred, however, the free observation was used to enable a greater knowledge and approximation through the interaction between the researcher and the research context.

Data collection was performed with or without interaction between nurse and parturient. In turn, the interactions varied in each woman participating in the study. The interactions were analyzed during the whole period of the active phase of the delivery, being concluded with the end of the active phase or when the woman was referred for cesarean delivery.

The data was organized in simple and cross tables. For the analysis of the proportions between Brazil and Cape Verde, according to the non-verbal communication of nurses and parturients, Chi-Square and Likelihood Ratio tests were used. For all inferential analyzes, those with p <0.05 were considered statistically significant. The study respected the Brazilian ethical precepts, according to resolution 466/12 of CONEP, with the approval of the ethics committee with the opinion 1,939,715. In Cape Verde, the legal and ethical precepts in force in the country were respected with the approval of the National Committee for Health Research based on the terms of article 9 of Decree-Law no. 26/2007.

## Results

Table 1 shows the number of interactions according to the non-verbal communication between the nurse and the parturient in both countries, in which there is a lack of interactions between nurses and parturients, prevailing in most variables , as for example in the analyzed distance. It is also observed that, in the last variables, the predominance of absence occurred in the interactions only in Brazil.

**Table 1:** Distribution of nurse-parturient interactions according to Non-Verbal Communication. Maracanaú, CE, Brazil and Praia, Santiago Island, Cape Verde, 2017

Variable	Brazil		Cape Verde		p
	n	%	n	%	
Nonverbal Communication-Nurse					<0.0001*
Nurse	258	43.7	67	56.3	
Absent	332	56.3	52	43.7	
Distance					0.005*
Intimate	175	29.7	37	31.1	
Personal	83	14.1	30	25.2	
Ausente	332	56.3	52	43.7	
Posture					<0.0001^†^
Standing	239	40.5	47	39.5	
Sitting	17	2.9	20	16.8	
Lying Down	1	0.2	-	-	
Kneeling	1	0.2	-	-	
Absent	332	56.3	52	43.7	
Axis					<0.0001^†^
Face to face	160	27.1	55	46.2	
Backwards	4	0.7	-	-	
Other angle	59	10.0	12	10.1	
Sociopeto-encouragement	34	3.4	-	-	
Absent	333	39.8	52	43.7	
Contact					<0.0001^†^
Touch	75	12.7	32	26.9	
Caring / Feeling up	14	2.4	-	-	
Grabbing	1	0.2	-	-	
Hold on for a long time	15	2.5	7	5.9	
Local touch	48	8.1	4	3.3	
Accidentaly graze	5	0.8	7	5.9	
No contact	100	16.9	17	14.3	
Absent	332	56.3	52	43.7	
Emblematic Gestures					<0.0001*
Tap the foot	7	1.2	1	0.8	
Move the hands	149	25.3	66	55.5	
Other	102	17.3	-	-	
Absent	332	56.3	52	43.7	
Illustrating Gestures					<0.0001*
Complements verbal language	177	30.0	65	54.6	
Does not complement	81	13.7	2	1.7	
Absent	332	56.3	52	43.7	
Regulatory Gestures					<0.0001*
Head shaking	97	16.4	1	0.8	
Move the eyes	74	12.5	66	55.5	
Others	87	14.7	-	-	
Absent	332	56.3	52	43.7	

* Chi-square test;

† Likelihood Ratio Test

Table 2 shows the number of interactions according to non-verbal communication between parturients and nurses from both countries.

**Table 2:** Distribution of interactions of parturients-nurses according to Non-Verbal Communication. Maracanaú, CE, Brazil and Praia, Santiago Island, Cape Verde, 2017

Variable	Brazil		Cape Verde		p
	n	%	n	%	
Nonverbal Communication-Nurse					0.018*
Parturient	262	44.4	67	56.3	
Absent	328	55.6	52	43.7	
Distance					0.003*
Intimate	177	30.0	35	29.4	
Personal	85	14.4	32	26.9	
Ausente	328	55.6	52	43.7	
Posture					<0.0001*
Standing	34	5.8	2	1.7	
Sitting	33	5.6	-	-	
Lying Down	186	31.5	65	54.6	
Posture	328	37.5	52	43.7	
Axis					<0.0001*
Face to face	159	26.9	34	28.6	
Another angle	59	10.0	33	27.7	
Ausente	328	55.6	52	43.7	
Contact					<0.0001^†^
Touch	28	4.7	16	13.4	
Grab	14	2.4	8	6.7	
Hold on long	8	1.4	15	12.6	
Accidentally brushing	48	8.1	8	6.7	
No contacts	148	25.1	20	16.8	
Absent	328	55.6	52	43.7	
Emblematic Gestures					<0.0001^†^
Move hands	129	21.9	67	56.3	
Other	131	22.2	-	-	
Absent	329	55.8	52	43.7	
Illustrator Gestures					<0.0001*
Complements verbal language	160	27.1	66	55.5	
Absent	329	55.8	52	43.7	
Regulatory Gestures					<0.0001*
Head shaking	110	18.6	2	1.7	
Move your eyes	84	14.2	65	54.6	
Others	66	11.2	-	-	
Absent	330	55.9	52	43.7	

* Chi-square test;

† Likelihood Ratio Test

Regarding the nonverbal communication of the parturient with the nurse, the absence of interactions in most variables also prevailed. It is important to emphasize that in the variables posture, emblematic gestures, illustrative gestures and regulatory gestures, there was a predominance of absence in the interactions also only in the Brazilian scenario.

## Discussion

Regarding the non-verbal communication of the nurse-parturient and parturient-nurse, it is important to note that, in the analysis of this communication, there was a greater absence in the interactions in Brazil.

Non-verbal communication, already studied by several authors and exposed in this study, enables people to express their feelings and emotions directly and has the functions of complementing, contradicting and/or replacing verbal communication^(^ 5 ^)^. The results show a considerable absence of non-verbal communication between nurse / parturient and parturient / nurse in the active phase of labor, a different reality found in Cape Verde.

The nurse professional should be available to this parturient offering support, security and effective communication. In both Brazil and Cape Verde, nurses, in some moments of interactions, have been directed to the use of cell phones and the Internet. Access to the cell phone and the internet can be a harmful factor in the communication process with this woman. Regrettably, with the insertion of the technological advance, mainly with the continuous use of the cellular apparatus and internet in work places, it is noticed that the commitment of the professional nurse, in some moments of his / her assistance to the patient, has been helped or prejudiced along years^(^ 9 ^)^.

The data from the study show that, in the two countries, the parturients established, in their interactions, nonverbal communication with the nurses (p = 0.018), presenting statistical difference. Interacting with the professionals who assist you in the pre-delivery gives the woman greater freedom and confidence in the team.

The woman needs to feel welcomed to communicate with initiative and freedom with the nurses who assist her in labor. Effective non-verbal communication implies valid and positive results both in the physical and psychological aspects of this woman^(^ 10 ^)^. In this perspective, the literature affirms that women in labor need to give birth in a place where they exercise total autonomy to verbally express their feelings^(^ 11 ^)^.

In relation to distance, the intima was predominant in both nurse-parturient communication (p = 0.005) and in the non-verbal communication of the parturient-nurse (p = 0.003). Intimate distance, in most assistance situations, is inherent to the nurse practitioner. The Ministry of Health (MH) advocates the approximation of health professionals in the care of their neighbors and bets on their participation in the improvement of this more humanized care^(^ 12 ^)^.

This approximation can be accomplished in the very exercise of care practices through simple interaction without requiring resources^(^ 13 ^)^. On the other hand, an “invasion” in the space of care can negatively affect the care process of women^(^ 14 ^)^. The absence of a spacious and individualized environment for each woman may have influenced the prevalence of intimate distance, although it may also have meant care and zeal.

As for the intimate approximation of parturients when interacting with nurses, it is noted that, in the majority of cases when there was non-verbal communication between these women and the Brazilian and Cape Verdean professionals, the women remained confident and confident in communicating with the recipient of the message for the time being.

In the axis variable, both non-verbal communication between nurse-parturient (p <0.0001) and between nurse-parturient (p <0.0001) had a predominance of the face-to-face axis. The professional interacts with the parturient, establishing face-to-face communication, provides greater bonding and security to this woman in normal birth^(^ 15 ^)^.

The woman also expects, from the obstetrician nurse, a face-to-face look at humanization and care for this phase. Therefore, all the circumstances that involve birth and birth may leave marks, positive or negative, unforgettable in a woman’s life^(^ 14 ^)^.

In the contact variable, there were also statistically significant differences between nurse-parturient (p <0.0001) and parturient-nurse (p <0.0001), in which the nurses in Brazil used no contact and touch, in Cape Verde, to establish interactions with parturients. The touch was considered as a variable of non-verbal communication and could be used to express feelings to the recipient of the communication. In the context of obstetric care, where empathy and contact are paramount, it is necessary to use this resource for non-verbal communication^(^ 8 ^)^.

With regard to women in labor, no contact was established in both countries. This event reflects on the possible lack of interest of these parturients in demonstrating tactile and close contact when interacting with this professional. During free observation, it may be noted that this absence may possibly occur through fear and fear. The contact between woman and professional team member should be evidenced in verbal and nonverbal communication between both. Postural, contact and distancing expressions may denote women’s insecurity and indifference towards the health professional in the care act^(^ 5 ^)^.

Regarding the social behavior of the nurse and the parturient, also evaluated in the study, this is understood through emblematic gestures, illustrative gestures and regulatory gestures.

In the emblematic gestures of nurses in both countries (p <0.0001), it was observed that professionals, in the course of the communication established with the parturients, moved their hands as a gesture and complement the verbal language that was transmitted to the women in active labor. The same happened to the Cape Verdean women (p <0.0001), because all the women in the parturients moved their hands. The characteristics of the emblematic gestures are present in the most varied cultures in the world. In the study, the parturients gesticulated with facial expressions in most of the interactions analyzed. This was due to the presence of pain and discomfort resulting from active labor. On the emblematic gestures of Brazilian women, there was a considerable percentage of other gestures in the interactions.

In relation to illustrative gestures, both nurse-parturient (p <0.0001) and parturient-nurse communication (p <0.0001) complemented the verbal language. To illustrate the communication with gestures becomes important, with a view to improving the understanding of the message transmitted to the parturient, especially in Cape Verde, where Creole exists as the mother tongue of almost all Cape Verdeans.

Most parturients, from both countries, supplemented verbal language. Thus, illustrative gestures are understood by imitation, accompanying speech, emphasizing the word or phrase pronounced by the individual^(^ 16 ^)^. Parturients should express themselves through gestures and / or speeches in the labor process. And free expression and autonomy is the right of every woman in the health services, and is indisputable in Brazil and in most other countries^(^ 17 ^)^.

Regarding the regulatory gestures, both in the nurse-parturient communication (p <0.0001) and in the parturient-nurse relationship (p <0.0001), the head nod was highlighted in Brazil, while in Cape Verde, eyes move prevailed. The nodding of the Brazilian nurses reinforces the speech of the other, and the movement of the eyes of the Cape Verdean nurses towards the woman in the parturient also reinforces the speech, whereas the deviation inhibits the same. These gestures aim to regulate and maintain non-verbal communication between people^(^ 18 ^)^.

Regarding the movement of the head and eyes of the parturients in communication with the obstetrician nurse, the importance given to this professional in the established communication stands out. Through regulatory gestures, these women expressed their interest in communication or their disapproval.

Next, the two variables that obtained different results between nurse-parturient and parturient-nurse communication, and that, secondly, were more prevalent in the study will be discussed.

When evaluating the nurses’ posture during the interactions (p <0.0001), the professionals remained standing during the established communication with the woman patient. It is natural, considering that the parturient is in the lying position, in the majority of the times, for the accomplishment of examinations, that the nurse adopts the standing position. The interactions occurred in different aspects, however, it was observed that most of the interactions occurred at the time of performing the vaginal touch tests for the verification of the uterine cervix dilatation and at the moment of the obstetric evaluation.

Differently from the reality experienced by the parturients about their posture (p <0.0001), the posture lying in both scenarios was predominant. In Cape Verde, women were little encouraged to stand or to be placed in another position during labor. As previously mentioned, the limitation of space is a factor considered. When they were encouraged to wander, most women rejected the suggestion provided by the nurses at the service. This rejection was due to the limitation of space in the environment and because this position is considered the most comfortable for women. It is also believed that the majority of Brazilian and Cape Verdean women preferred to remain lying down during the active phase of labor because they did not know the benefits of ambulation in active labor.

It is essential the preparation of these women for the normal birth still in prenatal care that attends the pregnant women. It is necessary to provide guidance and attention to the needs of pregnant women, by assisting them in relation to normal birth and other orientations of this important event^(^ 14 ^)^.

In Brazil, women were instructed by nurses to walk. While walking, the parturients interacted more with their companion. The benefits of walking to facilitate labor during parturition have been widely reported in the literature^(^ 19 ^)^. Stimulating and assisting the woman to walk can provide a more affective and close communication between the professional and the parturient, thus minimizing negative feelings that may be experienced by these women.

Anxiety, nervousness, sadness and fear are still feelings expressed by women in parturition process in several countries of the world. In this reality, when a parturient has indication for normal delivery and is admitted to a maternity hospital, it is still possible to find some routine procedures, such as venous access, enema, absolute rest in the bed, inadequate vaginal touch, childbirth in the lithotomic position, Kristeller maneuver, among others^(^ 20 ^)^. These procedures further increase these feelings of insecurity presented above.

In a study carried out in Nigeria, it was noted the extreme need to create public policies to support and humanize African women in the process of childbirth and birth. The results of the research suggest that the implementation of well-designed policies would certainly increase the quality and satisfaction of women at the birth of their children^(^ 21 ^)^.

In the Republic of Kenya, researchers investigated the satisfaction rate of women in labor. The main result of the study is that professionals working in obstetric care in the country are progressing in improving the care offered to women, but they need to move forward in the areas of effective communication, respectful and dignified care and emotional support during labor^(^ 22 ^)^.

In this context, working care and health promotion from the perspective of communication can modify the obstetric care framework. Attention should be paid to scientific and human care, since nurses and women in the parturition process should be informed about the importance of nonverbal communication as an effective strategy for the humanization of this care^(^ 23 ^)^, given that women are increasingly empowered and involved in their own parturition process^(^ 24 ^)^.

As limitations of the study, in Cape Verde, points to cultural and language issues in the country. Some parturients use only the Creole to communicate, thus causing difficulties in communication between the researcher and the participants at the time of the presentation of the study and invitation to participate.

## Conclusion

According to the analysis, all variables studied presented data with statistical difference (p <0.05). Intimate distance prevailed in interactions between participants in the two countries. The established posture between Brazilian and Cape Verdean nurses was the standing during the nonverbal communication established with the parturients. The study highlights that no contact was established by Brazilian nurses, while the Cape Verdeans used the touch to establish interactions.

During the interactions, the non-verbal communication between parturient and nurse was constituted by the intimate distance in the two countries. In the countries, it was found that the parturients remained lying during all non-verbal communication with the nurse. Parturients did not use contact to establish nonverbal communication with nurses in both scenarios. Similarities were observed in most of the analyzed variables of nurses and parturients in both countries. However, there were differences in some, such as in the posture of participants.

As it was concluded, in this study we see the relevance of non-verbal communication in the care process in the obstetric scenario in both countries, with a view to providing humanized and quality care. In this sense, it is suggested to emphasize the importance of the communicative process for this assistance. It is necessary to prepare these women for the parturitive moment so that they interact, in the best way, with the health team and vice versa. It is proposed, for future studies, the development of strategies to promote the acquisition of knowledge about non-verbal communication for obstetrician and parturient nurses in both countries.

## References

[1] Almagro JR, Martinez AH, Almagro DR, Garcia JMQ, Galiano JMM, Salgado JG (2019). Women’s Perceptions of Living a Traumatic Childbirth Experience and Factors Related to a Birth Experience. Int J Environ Res Public Health.

[2] Sanfelice CFO, Abbud FSF, Pregnolatto OS, Silva MG, Shimo AKK (2014). From institutionalized birth to home birth. Rev Rene.

[3] Jha P, Larsson M, Christensson K, Svanberg AS (2018). Fear of childbirth and depressive symptoms among postnatal women: A cross-sectional survey from Chhattisgarh, India. Women Birth.

[4] Melo DSA, Santos AA, Silva JMO, Sanches METL, Cavalcante KOR, Jacintho KS (2016). Woman’s perception on childbirth care. Rev Enferm UFPE.

[5] Rebouças CBA, Pagliuca LMF, Júnior JCR, Oliveira GOB, Almeida PC (2015). Comparative analysis of non-verbal communication between nurse and blind person. Index Enferm.

[6] Barboza LP, Mota A (2016). Obstetric violence- painful experiences among Brazilian laboring women. Rev Psicol Diversidade e Saúde.

[7] Abalos E, Oladapo OT, Chamillard M, Díaz V, Pasquale J, Bonet M, Souza JP, Gulmezoglu AM (2018). Duration of spontaneous labour in ‘low-risk’ women with ‘normal’ perinatal outcomes: A systematic review. Eur J Obstet Gynecol Reproductive Biol.

[8] Terra AC, Vaghetti HH (2014). Proxemics communication in nursing work: an integrative literature review. Cienc Enferm.

[9] Mesquita AC, Zamarioli CM, Fulquini FL, Carvalho EC, Angerami ELS (2017). Social networks in nursing work processes: an integrative literature review. Rev Esc Enferm USP.

[10] Dodou HD, Sousa AAS, Barbosa EMG, Rodrigues DP (2017). Delivery room: working conditions and assistance humanization. Cad Saúde Coletiva.

[11] Ribeiro JF, Machado PHF, Araújo KRS, Sepúlvedra BA (2016). Assistance under normal birth look parturient. Rev Eletr Gestão Saúde.

[12] Vogt SE, Silva KS, Dias MAB (2014). Comparison of childbirth care models in public hospitals, Brazil. Rev Saúde Pública.

[13] Silva FLF, Oliveira RCC, Sá LD, Lima AS, Oliveira AAV, Collet N (2014). Humanization of nursing care in a hospital environment: the user’s perception. Cienc Cuidado Saúde.

[14] Tostes NA, Seidl EMF (2016). Expectant mother’s expectations for birth and their perceptions of delivery and birth preparation. Temas Psicol.

[15] Côrtes CT, Oliveira SMJV, Santos RCS, Francisco AA, Riesco MLG, Shimoda GT (2018). Implementation of evidence-based practices in normal delivery care. Rev. Latino-Am. Enfermagem.

[16] Pagliuca LMF, Costa KNFM, Rebouças CBA, Almeida PC, Sampaio AFA (2014). Validation of the general guidelines of communication between the nurse and the blind. Rev Bras Enferm.

[17] Cortés MS, Barranco DA, Jordana MC, Roche ME (2015). Use and influence of Delivery and Birth Plans in the humanizing delivery process. Rev. Latino-Am. Enfermagem.

[18] Rebouças CBA, Pagliuca LMF, Júnior JCR, Oliveira GOB, Almeida PC (2015). Comparative analysis of non-verbal communication between nurse and blind person. Index Enferm.

[19] Souza SRRK, Gualda DMR (2016). The experience of women and their coaches with childbirth in a public maternity hospital. Texto Contexto Enferm.

[20] Lopes GC, Gonçalves AC Gouveia HG, Armellini CJ (2019). Attention to childbirth and delivery in a university hospital: comparison of practices developed after Network Stork. Rev. Latino-Am. Enfermagem.

[21] Okeke EM, Chari AV (2018). Health care at birth and infant mortality: Evidence from nighttime deliveries in Nigeria. Soc Sci Med.

[22] Oosthuizen SJ, Bergh AM, Pattinson RC, Grimbeek J (2017). It does matter where you come from: mothers’ experiences of childbirth in midwife obstetric units, Tshwane, South Africa. Reprod Health.

[23] Garnelo L, Lucas ACS, Parente RCP, Rocha ESC, Gonçalves MJF (2014). Organization of health care for chronic conditions by Family Health teams in the Amazon. Saúde Debate.

[24] Hidalgo-Lopezosa P, Hidalgo-Maestre M, Rodríguez-Borrego MA (2017). Birth plan compliance and its relation to maternal and neonatal outcomes. Rev. Latino-Am. Enfermagem.

